# Assessment of functional impairment after knee anterior cruciate ligament reconstruction using cardiorespiratory parameters: a cross-sectional study

**DOI:** 10.1186/1471-2474-15-163

**Published:** 2014-05-20

**Authors:** Marília Santos Andrade, Claudio Andre Barbosa de Lira, Rodrigo Luiz Vancini, Fernanda Patti Nakamoto, Moisés Cohen, Antonio Carlos da Silva

**Affiliations:** 1 Departamento de Fisiologia, Universidade Federal de São Paulo, São Paulo, Brazil; 2 Setor de Fisiologia Humana e do Exercício, Faculdade de Educação Física, Universidade Federal de Goiás, Goiânia, Brazil; 3 Centro de Educação Física e Desporto, Universidade Federal do Espírito Santos, Vitória, Brazil; 4 Departamento de Ortopedia e Traumatologia, Universidade Federal de São Paulo, São Paulo, Brazil

**Keywords:** Knee injury rehabilitation, Physiological responses, Heart rate, Oxygen uptake

## Abstract

**Background:**

A dynamic sub-maximum exercise with the same absolute intensity, performed with different muscle groups, may present exacerbated cardiorespiratory responses. Therefore, cardiorespiratory responses to unilateral exercise may identify bilateral differences. The purpose of this study was to verify whether the cardiorespiratory responses to lower limb exercise display counter-lateral differences, and if they could be used to assist athletes and health professionals involved in rehabilitation.

**Methods:**

Nine individuals participated in this cross-sectional study. They had been treated in a private rehabilitation clinic and submitted to intra-articular reconstruction of the anterior cruciate ligament. The cycling exercise with the same sub-maximal intensity and with one lower limb was used to gather data. Cardiorespiratory responses to exercise were compared between exercises performed with the involved and uninvolved limb after five minutes of exercise.

**Results:**

Cardiorespiratory responses to exercise performed with the involved limb presented higher values after five minutes of cycling: oxygen uptake (+7%), carbon dioxide production (+10%)_,_ minute ventilation (+20%)*,* breathing frequency (+19%)*,* ventilatory equivalent for oxygen (+14%), end-tidal pressure of O_2_ oxygen (+4%), end-tidal pressure of O_2_ carbon dioxide (-9%) and heart rate (+9%).

**Conclusions:**

The exacerbated responses, including increase of the ventilatory equivalent and decrease of end-tidal pressure of carbon dioxide_,_ indicate that this exercise protocol may be useful in the characterization of the functional deficit of the surgical limb during rehabilitation.

## Background

Knee anterior cruciate ligament (ACL) reconstruction is a common surgical procedure. In order to return to the original level of function as quickly and safely as possible, the patient must follow a rehabilitation protocol [1]. There is no agreement in relation to a optimal rehabilitation program following ACL reconstruction.

Recovery of the quadriceps muscle strength, power and endurance is among the main goals of the ACL rehabilitation programs because it is closely related to knee function [2]. Traditionally, muscle strength can be measured by manual muscle testing but this testing value is limited in detecting changes in strength [3-5]. Isokinetic dynamometers are also appropriate to detect changes in strength [6-8]. However, this equipment is expensive and its use during the initial months after surgery puts the patients at risk of exerting high levels of graft tension [9-11]. Thus, a safe method to assess exercise efficiency without increasing ACL graft load is required in the immediate post-operative phase.

In this context, a dynamic sub-maximum exercise with the same absolute intensity, performed with different muscle groups, may present exacerbated metabolic and cardiorespiratory responses. When the muscle group exercised presents lower functional capacity, the exercise will consequently be performed under higher relative physiological demand. Many authors reached that conclusion by contrasting arm cranking and cycling [12-14]; by comparing one to two legs cycling [15]; or when analyzing normal lower limb and lower limb with muscle atrophy [16]. However, these studies compared very different muscle mass, and we do not yet know if these cardiopulmonary-exacerbated responses can be observed when the exercises are undertaken with similar muscle mass, for instance, in a clinical post-surgical situation.

Therefore, the purpose of this study was to determine if there are differences in cardiorespiratory responses to exercise performed after unilateral ACL reconstruction, when the lower limb presents significant functional deficiency, at a constant submaximal intensity. We hypothesized that there are differences in cardiorespiratory responses to exercise performed with the involved limb when compared to the non-involved limb. This knowledge may contribute to the development of an objective physiological accessory criterion which would evaluate the existence of differences between limbs regarding functional status and muscle mass after surgery.

## Methods

### Participants

Nine participants (two women and seven men) volunteered to participate in this study. All participants trained at least for three days per week (3.5 ± 0.6 days per week), about 60 minutes per day, for at least one year before ACL lesion and they were sequentially invited to participate in the study, as they started the rehabilitation program, in a private orthopaedic clinic. The characteristics of the participants (mean ± standard deviation - SD) were as follows: age = 32.0 ± 1.4 years, body mass = 71.8 ± 10.8 kg and height = 172.5 ± 7.5 cm. The participants had ACL disruption and were submitted to intra-articular reconstruction of the ACL with patellar tendon graft by the same medical surgical team and participated in an intensive rehabilitation program in a private clinic. Briefly, the participants trained three times a week throughout the rehabilitation period and both limbs were treated. The assessment took place about two months after the surgical procedure (8.0 ± 1.3 weeks). The patients were informed about the purpose of the research and were asked to give their written consent to participate in a protocol approved by University Ethical Committee of Federal University of Sao Paulo. All procedures were in accordance with recommendations from the Declaration of Helsinki.

### Procedure

Participants were given a standardized set of instructions before performing the tests. The one-legged cycling exercise was performed on a bicycle (The Bike, Cybex, USA), with the patient in an upright position. On the first day, athletes started the pre-tests with the non-involved limb, with the aim of deciding the test absolute workload. Pre-tests consisted of some three minute exercise trials, in order to find the workload in which the heart rate (HR) was kept at 70% of the predicted maximum value (by 220 minus age), monitored using an HR monitor (Polar Electronics, Vantage, Finland). The same absolute workload was used in both limbs. On the second day, the exercise began with the involved limb. For rest measurements, the patient did not cycle for the first three minutes. This period was followed by cycling for five minutes with one limb. Afterwards, the patient recovered until the rest HR was reached. The same exercise protocol was repeated using the non-involved limb on the same day. During the test, respiratory gas samples were analyzed breath-to-breath using a portable metabolic system (K4b^2^, Cosmed, Italy) and each consecutive 20-second reading was averaged in order to smooth data. Before each test, the gas analyzers were calibrated according to the manufacturer’s recommendations. The variables measured were: oxygen uptake ($\dot{V}O_{2}$), carbon dioxide production ($\dot{V}CO_{2}$), minute ventilation (${\dot{V}}_{E}$), breathing frequency (*f*), tidal volume (V_T_), ventilatory equivalent for O_2_ (${\dot{V}}_{E}O_{2}$), ventilatory equivalent for CO_2_ ($\dot{V}\mathrm{EC}O_{2}$), end-tidal pressure of O_2_ (P_ETO2_), end-tidal pressure of CO_2_ (P_ETCO2_), and HR.

### Statistical analysis

Components used for sample size calculation were type I (α) or type II (β) errors. Type I was set as 5% and type II as 20%. For sample size calculation we used mean and SD values for HR and VE responses to exercise, extracted from similar previous studies, as it was recommended by Noordzij et al. [17]. The used values for VE calculation were: mean 1 = 98.13 L/min and mean 2 = 79.18 L/mim and SD = 14.0 L/min [18]. The used values for HR calculation were mean 1 = 213.8 bpm and mean 2 = 194.8 bpm and SD = 7.4 bpm [18]. The target number was 9 participants.

The last 20 seconds of exercise were used to determine data obtained from cardiorespiratory responses to exercise assessment. All variables presented normal distributions according to Kolmogorov-Smirnov tests. A paired Student’s *t*-test was used to compare variables obtained from the involved and non-involved limbs, and the level of significance was set at p < 0.05. In addition, we calculated the effect-size correlation values, using the means and standard deviations data. The results were expressed as mean ± standard deviation.

## Results

After five minutes of exercise under a constant submaximal intensity, significant increases were found in $\dot{V}O_{2}\left(+7\%\right)$, $\dot{V}CO_{2}\left(+10\%\right)$, ${\dot{V}}_{E}\left(+20\%\right)$*, f* (+19%)*,*${\dot{V}}_{E}O_{2}\left(+14\%\right)$, P_ETO2_ (+4%), P_ETCO2_ (-9%) and HR (+9%) values when the exercise performed with the involved limb was compared to that performed with the non-involved limb (Table 1). No significant differences were found concerning ${\dot{V}}_{E}CO_{2}$ values after five minutes of activity (p = 0.08).

**Table 1:** Physiological responses in non-involved and involved limb submitted to a cycling exercise with same absolute intensity

**Variable**	**Uninvolved limb**	**Involved limb**	**P**	**Effect size**
$\dot{V}O_{2}\left(\mathrm{mL}/\min\right)$	1680.4 ± 335.9	1802.7 ± 357.4*	0.02	-0.17
$\dot{V}CO_{2}\left(\mathrm{mL}/\min\right)$	1679.1 ± 259.4	1865.9 ± 314.8*	0.005	-0.30
$\dot{V}E\left(L/\min\right)$	54.1 ± 7.8	67.8 ± 15.4*	0.02	-0.49
Vt (L)	2.1 ± 0.4	2.2 ± 0.4	0.47	-
*f* (bpm)	26.4 ± 6.6	32.2 ± 9.6*	0.048	-0.33
$\dot{V}EO_{2}$	31.7 ± 7.4	36.7 ± 8.8*	0.047	-0.30
$\dot{V}\mathrm{EC}O_{2}$	31.1 ± 6.2	34.9 ± 6.1	0.08	-
PetO_2_ (mmHg)	99.6 ± 6.4	103.8 ± 5.8*	0.03	-0.32
PetCO_2_ (mmHg)	36.3 ± 5.7	33.0 ± 4.9*	0.04	0.30
HR (beats.min^-1^)	149 ± 16	162 ± 21*	0.005	-0.33

Data are expressed as mean ± standard deviation; *Statistically significant difference in relation to non-involved limb (p < 0.05). $\dot{V}O_{2}$: oxygen uptake, $\dot{V}CO_{2}$: carbon dioxide production, ${\dot{V}}_{E}$: minute ventilation, *f*: breathing frequency, V_T_: tidal volume, ${\dot{V}}_{E}O_{2}$: ventilatory equivalent for O_2_, $\dot{V}\mathrm{EC}O_{2}$: ventilatory equivalent for CO_2_, P_ETO2_: end-tidal pressure of O_2_, P_ETCO2_: end-tidal pressure of CO_2_ and HR: heart rate.

## Discussion

There are a lot of studies that aim to assess the ability of a patient to progress through the end stages of rehabilitation after ACL reconstruction [19-22]. However, it is very difficult to obtain safe and objective markers during the initial phases. In particular, the characterization of the functional level of the quadriceps is essential in order to determine an appropriate, low-risk increase in intensity of the rehabilitation load, considering the significant hypotrophy that this muscle presents after the surgical procedure, as well as its role in the stability of the knee joint [23]. Dynamic functional testing, for example the horizontal hop test, reproduces the functional movement, although its accuracy is somewhat questionable. Often, compensatory activities, such as swinging the arms, forward movement of the non-supporting lower limb or extending the trunk, mask deficits in the involved lower limb [24]. Furthermore, hop tests have been used only 4 or 6 months after surgery [20-22].

Other measurements can provide information about the deficiency in the involved lower limb. Thigh circumference is frequently used in the evaluation of muscle trophic conditions, although it underestimates the existing hypotrophy [25]. The Lysholm questionnaire can also be used to clinically evaluate knee conditions according to the patient’s perception [26].

Thus, in the present study, considering that the activity on the cycle ergometer is usually performed in the early stages of the rehabilitation procedure, we investigated whether the cardiorespiratory responses to exercise during dynamic constant submaximal intensity exercise in individuals that have undergone ACL reconstruction would be different between involved and non-involved limbs, two months after ACL reconstruction surgery. We found that cardiorespiratory responses obtained from exercise performed with the involved limb were exacerbated when compared with data from the exercise performed with the non-involved limb, at the same absolute workload.

In the current study, after five minutes of exercise in the cycloergometer, the $\dot{V}O_{2}$, $\dot{V}CO_{2}$, HR, and ${\dot{V}}_{E}$ presented higher values for the exercise with the involved limb. Such results probably reflect lower movement economy during the exercise performed with the involved limb as a consequence of poor oxidative status and lower involved muscle mass. The ventilatory effect was mainly determined by the breath rate, since the tidal volume did not vary significantly. The higher values of P_ET_O_2_ and $\dot{V}EO_{2}$ indicate that the ventilatory response per litre of O_2_ is increased in the exercise performed by the involved limb, representing a higher relative workload, although both limbs had worked under the same absolute workload. Some researchers also compared the cardiorespiratory responses to exercises performed with muscle groups of different functional capacities [13,27]. Likewise, the authors observed that in the same absolute workload there was an increase in HR and in $\dot{V}EO_{2}$ values during exercises that involved muscle groups submitted to a higher relative work intensity. Such effect is similar to that described by Wasserman et al. [28] for the incremental effort test and may indicate a major contribution of the anaerobic metabolism for the exercise with the involved limb. This analysis endorses the evidence that the same absolute workloads, applied to both limbs, represent a higher relative intensity for the involved limb, probably reflecting a functional deficit in the recovering limb.

In practical terms, as mentioned above, variables such as HR and *f* are exacerbated during the exercise performed with the involved limb, therefore monitoring these variables offers opportunities to directly evaluate the physiological profile of discipline-specific performance in an easy and inexpensive way. This suggests that health providers can use HR and *f* to form conclusions about the functional status of the involved limb.

Altogether, these findings corroborate previous studies regarding muscle strength deficiency after ACL surgery. Andrade et al. [25] showed that 4 months after ACL surgery, muscle peak torque deficiency in the quadriceps muscle was on average 46.7% and Strauss et al. [29] found 21.4%. Laffargue et al. [30] tested patients 6 months after surgery and found a quadriceps muscle deficit of 28.3%. However, despite the use of isokinetic muscle strength testing by clinicians and physical therapists to assess possible contra-lateral deficiencies and muscular balance, muscle strength testing conducted by isokinetic dynamometer may compromise the articular stability of the knee when applied during the initial months after surgery. Thus, the methodology proposed by this study could be an interesting alternative to evaluate and address the functional capacity of the muscle after ACL surgery. Furthermore, there are currently many studies investigating molecular composition (gene or protein levels) of the structures used for ACL repair in order to identify the best structure for a quicker rehabilitation [31]. Strategies to enhance the ACL healing process through overexpression of fibroblast growth factor (a powerful stimulator of fibroblast proliferation and type I/III collagen production) via direct recombinant adeno-associated virus vector–mediated gene transfer has also been investigated [32]. The results have a potential value for the development of novel and effective treatments for ligament reconstruction. Therefore, alternative measurements of functional evaluation after ACL surgery may also be useful to evaluate how these new interventions to accelerate ACL healing are effective in improving rehabilitation after ACL surgery.

### Limitations

In this study, we showed that after 2 months of a rehabilitation program following ACL reconstruction there are significant differences in cardiorespiratory responses to exercise when performed by the involved limb. However, we did not evaluate the how these bilateral cardiorespiratory differences changes with the muscular recovery during the rehabilitation phase until the patients are able to return to their normal sports activities. Another limitation may be the composition of the sample, which was taken by man and women with different ages. Another ligament injuries, besides ACL and the level of physical activity performed by the volunteers before the sugery also may be limitations of this study.

## Conclusions

Considering that both the $\dot{V}E$ and HR responses were exacerbated during the exercise performed with the involved limb, when a dynamic exercise, of constant workload and same absolute intensity (70% of the maximum HR during the exercise with the non-involved) was performed, the responses from the two variables could be measured to show incomplete muscle recovery of the involved limb.

### Clinical implications

Two months after unilateral ACL reconstruction there are differences in cardiorespiratory adjustments, such as HR and *f*, to dynamic exercise performed by uninvolved or involved lower limbs. These findings can contribute to the development of an easy and inexpensive rehabilitation monitoring protocol, with physiological bases, which may show the evolution of the patient over the initial months of rehabilitation after ACL reconstruction, taking the non-involved limb as reference.

## Abbreviations

ACL: Anterior cruciate ligament; f: Breathing frequency; HR: Heart rate; PETCO2: End-tidal pressure of CO_2_; PETO2: End-tidal pressure of O_2_; ${\dot{V}}_{E}$: Minute ventilation; $\dot{V}\mathrm{EC}O_{2}$: Ventilatory equivalent for CO_2_; ${\dot{V}}_{E}O_{2}$: Ventilatory equivalent for O_2_; $\dot{V}O_{2}$: Oxygen uptake; $\dot{V}CO_{2}$: Carbon dioxide production; VT: Tidal volume.

## Competing interests

No commercial party having a direct financial interest in the results of the research supporting this article has granted or will grant any benefits to the authors or any organization with which the authors are associated.

## Authors’ contributions

MSA: study concept and design; data acquisition, analysis, and interpretation; manuscript preparation and critical revision of the manuscript. CABL: study concept and design; data acquisition, analysis, and interpretation; manuscript preparation and critical revision of the manuscript. RLV: data analysis, interpretation, manuscript preparation, and critical revision of the manuscript. FPN: data analysis, interpretation, manuscript preparation, and critical revision of the manuscript. MC: study and conception, manuscript preparation, and critical revision of the manuscript. ACS: study and conception, manuscript preparation, and critical revision of the manuscript. All authors read and approved the final manuscript.

## Pre-publication history

The pre-publication history for this paper can be accessed here:

http://www.biomedcentral.com/1471-2474/15/163/prepub
